# Intelligence

**Published:** 1828-08

**Authors:** 


### INTELLIGENCE.

### MONTHLY REPORT OF PREVALENT DISEASES.

NOTWITHSTANDING the variable state of the weather throughout the whole of the last month, no serious disease has prevailed. Cholera has not been unfrequent: it has been mild in its attack, and of short duration, in all the cases we have seen. Slight bronchial affections have also been common. Asthmatic patients have suffered severely, as might be expected from the dense state of the atmosphere. An interesting case of complete paralysis of the lower extremities of a child has occurred to us. The digestive organs were much deranged, and in about ten days after active purgatives had been given the little patient was completely restored to health. In one case of inflammation of the bowels, the benefit of full doses of opium, after free bleedings, was clearly shewn.

---

*Royal College of Surgeons.*—Sir ANTHONY CARLISLE has been elected President; H. L. THOMAS, Esq. and Sir P. M'GREGOR, Vice-Presidents; and J. BRIGGS, Esq. a member of the Council.

Mr. GUTHRIE is appointed Professor of Human Anatomy and Surgery, and Mr. HERBERT MAYO Professor of Comparative Anatomy.

---

We have to announce, with very sincere regret, the death of Sir P. M'GREGOR, Serjeant-Surgeon to the King, &c. &c. But a few days before his decease, he had been elected Vice-President of the Royal College of Surgeons. Eulogies are not uncommonly lavished upon the dead, as a mere matter of form; but of Sir Patrick M'Gregor it may be said, with the strictest truth, that he was universally respected, as well by the public as by his professional brethren.

---

*Literary Notice.*—We have received the third edition of Dr. GREGORY'S "Elements of the Theory and Practice of Physic." The whole has been carefully revised. Several topics, unnoticed in the preceding editions, have been introduced, as Delirium Tremens, Paralysis Agitans, Cachexia Africana, Hepotalgia, &c. In 1826, this work was published at Philadelphia, with very copious notes and additions by Professor POTTER, which have been incorporated with the present edition by Dr. GREGORY. We have before stated our favorable opinion of the work. It is decidedly the best on the subject in the English language.

---

Mr. BROOKES'S *Museum.*—THE sale of this splendid collection is still continuing, and many of the choicest anatomical preparations, and specimens of morbid anatomy and natural history, are yet to be disposed of. Those anatomical preparations which were known to have been dissected by Mr. BROOKES have been eagerly sought after, and have generally brought high prices. Mr. CLIFF has been a constant attendant, and the College of Surgeons has purchased freely and liberally. A very spirited competition took place for the skeleton of the Peruvian Paco, a most beautiful animal, between the College and Mr. TEMMINCK, who has been sent over expressly for the purpose of enriching his national collections, by the Dutch government. It was sold to the College for thirty pounds. It is said to be the only skeleton of this animal in Europe. The Chilian Lama, presented to Mr. Brookes by Lord Darnley, produced twenty-six guineas. This lot was also bought by the College.

With every true lover of his profession and of science in general, it must be a subject of deep regret that the value and interest of *such* a collection should be diminished by separation. Many reports have been circulated of handsome offers having been made for the whole museum, on the part of government and different universities. They are, however, without foundation. We know, from the best authority, that no attempt even has been made to secure this invaluable collection as a national museum, or to promote the cause of science by attaching it to any university.

---

*To the Editor of the London Medical and Physical Journal.*

Sir,—It has been recently much the fashion to decry, for party purposes, the education which the English Universities require for their graduates in medicine.

After the same education which is demanded from persons qualifying for the learned professions, as the church or the bar, a sufficient time elapses before the first medical degree is conferred, to enable them to acquire (with powers of mind reasonably believed to be improved by such previous education,) medical science at any of the universities on the continent, or in Edinburgh or in London, as they please. The test of their proficiency is an examination, without passing which they are not admitted to their first degree in medicine; a degree, be it observed, which does not even authorise practice until a licence is given *ad practicandum*, and which is wholly unavailing in London until the possessor of it has been examined and licensed by the London College.

The following are the questions set to the candidates for the degree of B.M. in the University of Cambridge, in June last. The public will judge whether such an examination is inferior to any in Europe in difficulty, and whether persons answering them fully on paper are not qualified for admission to their first degree.

I am, sir, your obedient servant, VERAX.

*Examination for M.B. Degree.* 1828.

No. I.

1. Describe the Cæliac Artery, its branches, and their distribution.
2. What are the branches of the External Carotid Artery?
3. Describe the Sinus' of the Brain, their form, situation, and structure.
4. Describe the origin, course, and distribution of the Par Vagum.
5. What are the Nerves distributed to the muscles of the face? What is the difference in function attributed to the fifth and seventh pair?
6. Describe the Pericardium, its situation, attachments, structure and use.
7. Describe the Omentum, its form, attachments, and position. What is meant by the small Omentum?
8. Describe the Duodenum, its position, attachments, and structure.
9. What are the changes which the Blood and Air undergo in respiration? Is the Circulation assisted by atmospheric pressure?
10. What is the chemical composition of Bile? What purpose does this fluid serve in the animal economy?
11. For translation, Aphorisms from Hippocrates.

**No. II.**

1. What are the morbid appearances found on the dissection of persons who have died from Apoplexy?

2. Explain the Pathology of Dropsy. In what cases is bleeding to be recommended in this disease?

3. In what stage of Measles does diarrhœa usually occur?

4. What remedy does Sydenham recommend in the diarrhœa supervening on Gout?

5. What is the distinction between erythematous and phlegmonous Inflammation?

6. What are the symptoms and treatment of Tetanus?

7. In what class and order of Cullen's Nosology is Dyspepsia placed?

8. How do we distinguish Pleurisy and Peripneumony?

9. What are the symptoms and treatment of Cholera Morbus? How do we distinguish this disease from the effects of the swallowing of arsenic? What are the best tests of the presence of the arsenious acid?

10. What are the mode of preparation, the dose, and the medicinal powers of the Bizmuthi Subnitras?

How is the liquor Ammoniæ prepared? What are the medical virtues and dose of this preparation? What is the chemical composition of Ammonia? What is its equivalent number?

How is the Acetas Plumbi prepared? What are the medical virtues and dose of this preparation?

What is the formula for the liquor Plumbi Subacetatis dilutus?

What is the mode of preparation and dose of the Infusi Digitalis? With what medicines is it incompatible?

To what class and order of Linnæus does Digitalis belong? To what natural order?

11. For translation, a passage from Celsus.

---

### THE COLLEGE OF PHYSICIANS *v.* HARRISON.

We are indebted to a friend for the short-hand writer's report of this important Trial, which has been procured at considerable expence: we are thus enabled to give a much more correct account of it than has yet been published. The opening speech of Sir J. Scarlett, and Mr. Campbell's defence, are copied verbatim. We have passed over the recapitulation of the evidence contained in the summing-up of Lord Tenterden; and, as a considerable portion of the evidence itself is not much *ad punctum*, we have taken only the substance of it. The whole of the correspondence between Dr. Harrison and Dr. Chambers, and the College of Physicians, has already been given in our Journal.*

*Court of King's Bench, Westminster, July* 3, 1828.

*Before the Right Hon.* Lord Tenterden, *and a Special Jury.*

Mr. Parke opened the pleadings.

Sir James Scarlett.—May it please your Lordship; Gentlemen of the Jury; This is an action brought by the President and Fellows of the College of Physicians, for the purpose of enforcing a regulation made by a statute as

* Vide Number for July 1827, p. 85 et seq., and October Number, p. 375 et seq.

early as the time of Henry VIII., and which is incorporated in their charter, for the purpose of preventing persons who have not been duly licensed by the College from practising in London, or within seven miles of it.

Gentlemen, it is quite unnecessary that, in enforcing a penalty of this sort, I should enter before you into a discussion of the policy of it, because the only question will be whether, in point of law, the College of Physicians have a right to enforce that penalty. Whether or not it was expedient in King Henry VIII. to grant a charter to the College of Physicians,—whether or not it was right for Parliament to confirm that charter, which they did some time in the reign of the same king,—are questions with which we have nothing to do. I am more desirous of saying this in the outset, because, in the course of my experience in this place, I have several times had occasion to observe that very often the attention of juries is misled, and very often an attempt is made to mislead the attention of judges, from what is the real law of the land to what is the policy of the law, with which in this case we have really nothing to do. I shall, therefore, not occupy any time in vindicating that policy by which the Parliament attempted, in the time of King Henry VIII., to prevent the public from becoming a prey to empirics and quacks, who were not duly qualified to practise the science of physic. Whether the gentleman who is the defendant in this case is highly gifted in his profession, or whether he is deficient in the qualities which are necessary to practise that profession, highly useful and highly honourable as it is, is not the subject of inquiry; for every one must feel that, if the College of Physicians should relax from their duties and the observance of their rules, out of respect to any individual, however highly gifted, it would follow they would expose themselves to the charge of the grossest partiality, and it would be said of them, and with justice, that they make selections, and allow persons to practise from favor and interest, preventing others, and that the mode of accomplishing the objects of this statute, which was undoubtedly to prevent persons not wanting skill from practising, was to enforce the rules generally against all. The rule is, that no person shall be permitted to practise in London, or within seven miles of it, unless he shall be examined by the College of Physicians, according to the regulations they make, and found by them competent to practise. There are certain exceptions made, which do not affect this question, in favor of the graduates of the English Universities. Those exceptions relate to practising at a greater distance from the metropolis, and have nothing to do with this question; but, with regard to the case now before you, it turns upon the charter of the College, confirmed by the act of Parliament, which declares it shall not be lawful for any man to practise physic in the metropolis, or within seven miles of it, unless he shall previously submit to an examination by the College, and receive a certificate under their seal for that purpose.

Gentlemen, Dr. Harrison, the defendant, has for some time been, as we are informed, practising as a physician. I should have stated to you that the charter requires that the practice should be for one month,—that is to say, he should be in practice for one month,—because an occasional advice to a friend, or a prescription which a gentleman gives who comes from the country, would not be considered a violation of this statute; and, therefore, the regulation requires that a person should practise for one month, —that is, that he should not practise every day for a month, but should hold himself out and practise as a physician for a month. The time that is necessary for enforcing the penalty throws very often some difficulty in the way of the proof, because, as a physician practises amongst his own particular class of patients, it is not always, nor is it often possible, to discover among them a disposition to communicate evidence with respect to his practice.

It so happened that a considerable time ago—a year or more ago—a member of the College of Physicians had occasion to observe Dr. Harrison in practice, and some correspondence took place between them in consequence of that; because, gentlemen, in obedience to a bye-law of the College, refusing to consult with him as a physician because he was not licensed,—not out of any disrespect to Dr. Harrison, but because every physician is liable to a fine, by the bye-law, if he consults with any one who is not licensed by the College. That produced an anxiety on the part of Dr. Harrison to meet the question fairly. He directed a letter to the College, in which he states an objection upon the act of Parliament, which is founded upon a mistake, for want of advice; because I am sure his learned counsel will not insist upon it, as his objection upon the act of Parliament is a frivolous objection; for he says there are no censors. They do not want censors; and therefore the objection is a frivolous objection, and shews the Doctor is ignorant of the law more than of his own profession. But, when the Doctor was desirous of undertaking this contest, it became necessary for the College to meet this gentleman, and he was summoned to attend them. He declined to attend; but avowed his intention of meeting them fairly in a court of justice. It was then necessary to inquire what evidence there was that he had practised for a month; and the Doctor wrote a letter, which I will read to you, addressed to the College of Physicians, in reply to their last requisition that he should attend at their board, and be licensed, if found duly qualified.

Dr. Harrison's letter (No. 8,) to the Censors of the College was read.

Well, gentlemen, upon that letter being received, it was thought that the plaintiffs could do no less than apply to Messrs. Tennant and Harrison, to know whether they would admit that the Doctor practised for a month, in order to raise the question. They took time to consider, and then returned for answer they could make no such admission; so that this profession which the Doctor made that he would discuss the matter fairly, was defeated by the caution of his solicitors,—I will not say, prompted by him, but by the usual caution of lawyers, who will not admit any thing; and therefore the College were obliged to procure evidence to put this matter fairly at issue. They have done that. They would have been better pleased if the Doctor had made a fair admission; but, finding he would not do so, in order to gratify him in raising the question, they have sought for other evidence, and that evidence will be produced to you. I need not state the evidence; but I think we have prescriptions from the month of April to the month of November, 1827. I do not mean to enter into any critical discussion of the nature of the prescriptions; that is not for you to decide; and therefore I shall say nothing about that. They are sufficient to show the Doctor prescribed as a physician; and this letter, and others, will shew that he avowed that he prescribed as a physician; and I believe I can shew that one lady, whom he attended for one or two years,—for the Doctor does not pursue his profession without profit;—I can shew that he received in the course of two years 500 guineas, and therefore he could not say that he practised gratuitously; but he is strictly within the definition of a physician practising for emolument, which he has a right to do by the law of the land. Now, gentlemen, I do not put in issue the fitness or propriety of Dr. Harrison; that is not the issue; and I should be extremely sorry to cast any reflection upon that gentleman. I do not put it upon that ground. The question must be treated in the same way as if it was Sir Henry Halford who refused to submit to the regulations of the College, or if it was the veriest quack, Dr. Solomon or Dr. Brodum; and therefore those are not the questions. Those would be the questions if they came to be examined. It certainly might be a presumption, though I do not press that against this gentleman, that when he refuses to submit himself to the examination of impartial and competent men, that it is a presumption against his competency.

Gentlemen, I shall put in this act of Parliament, which is known to his lordship being upon the statute-book. It contains the charter, and embodies the whole of it, and his lordship will recollect that was recognised in Bonham's case, which was tried before the Court of Common Pleas, in the time of Lord Coke, and therefore there will be no question about that. We shall produce evidence to shew he has practised for a month, and we shall be entitled to ask of you the penalty of five pounds for each month.

*Evidence for the Plaintiffs.*

Dr. Turner was sworn. His evidence was objected to by Mr. Campbell, who "apprehended he had a clear interest in the event of the suit," as he was a Fellow and the Treasurer of the College of Physicians.

Sir James Scarlett replied, that "this was the first specimen of a fair discussion of the question." The evidence of Dr. Turner was not continued.

Mr. J. Roberts, clerk to the College, produced the charter of the 10th of Henry VIII.

*Mr. Brougham.*—Your lordship will find it is set forth in the preamble of the statute.

*Lord Tenterden.*—Is it set forth word for word in the act of Parliament?

*Mr. Brougham.*—Yes, my lord.

*Lord Tenterden.*—It is set forth verbatim in the act of Parliament.

*Mr. Campbell.*—At present, my lord, I do not allow that it is an act of Parliament.

*Lord Tenterden.*—That is a question that will be more fitly discussed in another Court.

*Mr. Campbell.*—My lord, I submit it must be discussed here.

*Lord Tenterden.*—Am I not doing better for you by reserving the point?

*Mr. Campbell.*—My lord, unless my friends produce better evidence of an act of Parliament than the existence of an act in an edition of the statutes, I shall submit the plaintiffs must be nonsuited.

*Lord Tenterden.*—Do I not do better for you by reserving the point?

*Mr. Campbell.*—I should wish your lordship to direct the plaintiff to be nonsuited.

*Lord Tenterden.*—Prima facie, a statute being to be found is evidence that it passed.

*Mr. Campbell.*—My lord, I should submit, if it had the royal assent, I should say this was no evidence; and, unless there be a copy produced from the Parliament rolls, this important allegation in the declaration is not proved.

*Lord Tenterden.*—Then we will read the charter.

*Mr. Campbell.*—To facilitate the progress of the cause, there is no objection to reading it as from a copy.

*Sir J. Scarlett.*—The assumption here is, that it is a private act of Parliament. That is the assumption of my friend.

*Lord Tenterden.*—I will listen to any argument with great pleasure, Mr. Campbell; but you must be sensible the question whether a document purporting to be a statute, be or be not an act of Parliament, and be or be not a private act of Parliament, is a point of much too great importance for a single judge to decide.

*Mr. Campbell.*—If the judge entertains doubt upon it, undoubtedly; but I trust, with the assistance of my friend Mr. Armstrong, I shall be able to remove any doubt your lordship may entertain upon it.

*Lord Tenterden.*—But I think you can hardly suppose any decision I come to will be satisfactory. Whichever way I decide, the other party will apply to the Court. You will have the benefit of the opinions of all the four judges, whichever way I decide. We may read the charter from the act of Parliament.

*Sir James Scarlett* read the passage in the charter upon which the proceedings were founded.

*Mr. Campbell* replied—I will point out what seems to be an objection to the declaration. The declaration alleges that King Henry VIII., by his charter, "did, amongst other things, grant to certain persons therein named and expressed, that they should be, in deed and in name, a body and perpetual commonalty, or a perpetual college." He granted to the persons therein named and expressed, that they (that is, the persons therein named and expressed) should be so. Now, I will point out to your lordship what seems to me to be a fatal variance, because there is "memoratis doctoribus Joan

Chambre, Thomas Linacre, Ferdinando de Victoria, medicis nostris, Nicholao Halsewel, Joanni Francisco, et Rob Yaxley, medicis concessimus, quod ipse omnesque homines ejusdem facultatis de et in civitate prædicta, sint in re et nomine unum corpus et communitas perpetua sive collegium perpetuum." Now the grant is, not the six persons named Chambre, Linacre, Ferdinand de Victoria, Halsewell, Francis, and Yaxley, were to become a perpetual commonalty and college, but that they "et omnesque homines ejusdem facultatis,"—that they, and all the men of the same faculty, were to become so. Now, my lord, I believe the College have got a wrong copy of their own charter, and that they have acted upon it as if the charter were a grant to these six individuals, and such persons as they might choose to be their successors, and not to the "omnesque homines ejusdem facultatis;" because I believe what they have done is to refuse to admit "omnesque homines ejusdem facultatis," although a person may be as learned as Sir Henry Halford himself, and may have taken his degree at Padua or Edinburgh, or the most learned school in medicine. They will not admit him; and they proceed upon the supposition that there are no such words in the charter. Now I submit to your lordship, these are words in the charter, and most material words, and this grant is not by Henry VIII. to particular individuals named only, but to all men of the faculty of medicine: that is, to all who have regularly graduated at any acknowledged university. That being so, my lord, I submit to your lordship, that this allegation in the declaration that there is that grant to the persons named, and to them only, is not substantiated; and upon that ground the plaintiffs should be nonsuited.

*Sir J. Scarlett.*—My lord, my friend's objection divides itself into two parts: one is a mere formal objection to the declaration, on the ground of variance between that and the charter produced; and the other is a discussion merely of the merits, whether or not the charter can receive the construction which is put upon it by the College of Physicians; which last question I beg to postpone.

*Lord Tenterden.*—That question has nothing to do with it.

*Sir J. Scarlett.*—Then, my lord, the question is, the charter constitutes individuals who are named, and the then medical practitioners and graduates of the universities of England, a corporation. My friend says that the declaration does not, in the description of the charter, give it to all the body, but confines it to the individuals named. It will be for your lordship to say whether the words of the declaration will bear that interpretation. Now the words are, that King Henry VIII. granted to certain persons, therein named and expressed; and the question will be, whether the word expressed will not sufficiently embrace all the other persons who were not named specifically, that are expressed and declared to be the then persons practising medicine? I apprehend, my lord, that is sufficient, that it is not such a variance as is suggested. If this declaration had stated a grant to a larger body than the charter implies, that would have been a variance. There is no doubt that the grant was to the individuals named; but, if it be necessary also to say, besides the individuals named, there was a class of persons not named who were made members of the corporation, I submit that the word expressed is sufficient to shew that the charge of variance is not justly ascribed to this declaration. I should state to your lordship, this follows a precise precedent of a similar proceeding which has taken place before; and therefore, though I am not answerable for the declaration, my friend, who drew it, made it like a precedent which took place before.

*Mr. Brougham.*—Your lordship sees the persons therein named are those very persons, and the persons expressed are those who practised medicine.

*Lord Tenterden.*—Now, Mr. Campbell.

*Mr. Campbell.*—The question is, whether expressed means implied? Now I always thought expressed had been opposed to implied. My friend says, by expressed must be meant those that are not named. Now I say, expressed goes on to express more specifically what it is to those who are named and expressed; and you can never, under the word expressed, say it is granted to any persons by implication. Now, my friend Sir James Scarlett says, discussing that objection, if there were others to whom it was granted, it would be no fatal variance. My lord, I apprehend that is clearly untenable; for suppose it had been to these persons and others not named, there was a body totally distinct; and, if it had been excluded by the manner in which the grant is stated in the declaration, I apprehend that would be a fatal variance; and the question is whether, by the word expressed, you can include those who are not expressed.

*Lord Tenterden.*—I am very clearly of opinion that the word expressed in this declaration means, omnesque homines ejusdem facultatis; and therefore there is no variance.

*Mr. Campbell.*—Very well, my lord.

*Lord Tenterden.*—Then now, I suppose, you will direct my attention to the statute contained in your declaration.

*Sir J. Scarlett.*—Yes, my lord. It is the 14th and 15th of Henry VIII. c. 5. It was recognised by several subsequent statutes.

*Lord Tenterden.*—Now arises Mr. Campbell's objection.

*Mr. Campbell.*—If my learned friends say, by putting that printed book in your lordship's hands, that would have satisfied the allegation in the declaration that this charter was confirmed by act of Parliament, then I say it is no evidence, and the plaintiffs must be nonsuited. It is quite clear there must be a confirmation by act of Parliament; for the king, by his royal prerogative, could not have any power to create a body, and give a right to levy a penalty upon the subject. It may be done by act of Parliament. Now, there is an allegation that the said letters patent—

*Lord Tenterden.*—Were confirmed by act of Parliament.

*Mr. Campbell.*—Were confirmed by the statute in that case made and provided. The evidence of that is, to hand up to your lordship a printed book professing to contain the statutes of the realm. Now my first objection is, that even if that were upon the face of it an act of Parliament confirming that charter, that it would not be admissible evidence for the purpose. This is a private act of Parliament: it relates to a particular class of his Majesty's subjects, and to a particular place,—to physicians within seven miles of London; it is therefore a private act of Parliament, and a private act of Parliament is not to be proved by a printed collection of the statutes. It must be proved by an examined copy from the Parliament rolls. Now, secondly, suppose that an examined copy from the Parliament rolls of that which lies before your lordship were produced, then I say that would not prove that any such statute ever passed; for it is only a document merely in the form of a petition from those persons named in the charter, that it may please the king that such and such a statute may pass. Now, there is nothing there of "le roi le veut," or any thing to intimate that the royal assent was given to the petition. If a witness were called from the Tower of London, who said this petition was found in the rolls, and that there were the words upon the roll "le roi le veut," that might be evidence to be received that an act of Parliament did pass both houses of legislature, and did receive the royal assent; but there is no evidence to shew it ever passed the commons' house, it ever passed the lords' house, or received the royal assent; and, therefore, if it were produced from the Tower of London separately, they would prove nothing: and on both these grounds I submit the plaintiffs must be nonsuited.

*Sir J. Scarlett.*—My lord, my friend is too severe with me.

*Lord Tenterden.*—Does Mr. Armstrong wish to add any thing?

*Mr. Armstrong.*—I would merely add, that it is usual, even in public acts of Parliament, to have a clause to make them evidence; without which they are not evidence.

*Lord Tenterden.*—There is no doubt as to the principle: the only question is as to the application. As at present advised, I think this is a public act, which applies to all London, and the last section, which applies to the whole kingdom: but I will save that point, Mr. Campbell. I cannot do better for you than that.

1

*Sir J. Scarlett.*—It is for the health of all his Majesty's subjects.

*Lord Tenterden.*—You will move for a nonsuit, Mr. Campbell.

Emma Edwards, sworn.—Her evidence proved that Dr. Harrison had attended her mistress, Miss Orton, for a considerable period, and that he received fees in the usual way. She had frequently seen Dr. Harrison write prescriptions. She identified those which were handed to her by counsel as having been written for her mistress by Dr. Harrison. They were made up at Mr. Yarde's, the chemist. Dr. Harrison had also attended witness herself. Miss Orton was not able to attend the trial.

*Cross-examined by Mr. Campbell.*—Q. You were the servant of Miss Orton? —A. Yes.

Q. I believe this young lady unfortunately had an affection in the spine?— A. Yes.

Q. A curvature in the spine?—A. Yes.

Q. Was it not for that that Dr. Harrison attended her?—A. Yes; and *for the health as well.*

A. Her health was affected from this affection of the spine, was it not ?—A. That I do not know.

Q. She had this affection of the spine during all the time Dr. Harrison attended her, had not she?—A. Yes.

Q. Were you ever in the room when means were taken to cure this curvature?—A. Yes.

Q. Was not that done by the hand?—A. Yes.

Q. By friction, by rubbing, and by trying to lengthen the spine?—A. Yes.

Q. Was he not generally half an hour employed in these operations?—A. Yes.

Q. Trying by the hand to strengthen the spine?—A. It was done by extension.

Q. Mechanical means were employed?—A. Yes.

Q. *(by Lord Tenterden,)* By the hand, or was an instrument employed?— A. An instrument was employed, I believe.

Q. *(by Mr. Campbell,)* By the hand and by an instrument?—A. Yes.

Q. Was not that done almost every time that Dr. Harrison came?—A. Yes.

Q. You say that Dr. Harrison gave you some advice?—A. Yes.

Q. He did not take any thing from you, I suppose?—A. No.

Dr. Harrison's letter, No. 8,* was again put in, and read.

Mr. Murrell Pickthorn, sworn, (*examined by Mr. Parke.*)—Deponent was a surgeon and apothecary. He now attended Miss Orton. Had made up the prescriptions of Dr. Harrison. They were for internal remedies. Had also made up prescriptions of Dr. H.'s for Emma Edwards. They were also internal remedies.

Mr. J. Roberts, recalled.—Had applied to the attorneys of the defendant for admission that he had practised as a physician. They consequently applied to the defendant, who declined making any admissions.

*Sir J. Scarlett.*—I am going to put in another letter, which I did not mean to have read, but in order to allay any question as to his practising as a physician.

Dr. Harrison's letter to Dr. Chambers† was then read.

Mr. Campbell.—May it please your Lordship ; Gentlemen of the Jury : I do confess I am a little surprised that this learned body should come into his Majesty's Court of King's Bench with such a case as my learned friend, Sir James Scarlett, has presented before you. I might call upon my Lord Tenterden, I apprehend, to say there is no case for your consideration at all.

* London Med. and Phys. Journal, October 1827, p. 377.

† Ibid. July 1827, p. 85.

But, however, gentlemen, I will confess that there is some evidence, however slender, for your consideration; but you, having considered that, I feel the utmost degree of confidence, will find a verdict for my friend.

Gentlemen, I begin by disclaiming, on the part of my client, any irritation, any ill will, any ill opinions of the individuals of whom the Royal College of Physicians is composed; and, above all, to remove any imputation from the character of the learned president, Sir Henry Halford, for whom my client, Dr. Harrison, who sits before me, along with the community at large, entertains the utmost possible respect and regard. He is a most learned physician, —he is a most honourable man,—and I rather suspect that, if he were in court, he would be very much ashamed of this proceeding, which has taken place in the name of the body over which he presides. But while I mention with regard and honour the individuals of whom the College of Physicians is composed, I must speak of them as a body, upon this occasion, with indignation and reprobation. I think their conduct is illegal. I think that it is disgraceful. They are attempting to deprive his Majesty's subjects of that skill and that attention which may be devoted to their relief and the improvement of their health.

Gentlemen, I should have thought that these were not exactly the times to bring forward an obsolete act of Parliament, passed in arbitrary times. This charter by itself is contrary to law. I respect the prerogative as much as any man, but I must point out the limits of that prerogative. The king has no right to create a body with a right to levy a penalty upon any of his subjects. Such was the charter made under the mild government of Cardinal Wolsey; and, for five years after that charter was granted, it remained without any act of Parliament, and it was not until the fifteenth year of the reign of Henry VIII. that, under the same auspices, a pretended act of Parliament is supposed to have passed, about which we may hear, if necessary, another day, —but a question which I think probably never will arise,—whether it was sanctioned by the legislature or not. But be it sanctioned, or be it not sanctioned, by the legislature, gentlemen, I think the College of Physicians would have acted more prudently, more discreetly, if they had kept it quietly in their recesses, instead of blazening it in open day, and exposing it to the public gaze.

Now, gentlemen, what is their charter, and what is this act of Parliament? I say, gentlemen, I will not, as my friend Sir James Scarlett says, enter into the policy of it. I will suppose it was most legal, most politic, most wise. The question you have to determine, I allow, is, has this charter, and has this act of Parliament, been violated? Has there been evidence laid before you to shew that my client Dr. Harrison has incurred these penalties?

Now, gentlemen, you will observe that the penalty is this, for having practised as a physician,—for having practised the faculty of physic for a month continuously; and unless Dr. Harrison is proved, in medical cases not surgical,—not where he is using his hand,—not where he is using mechanical means, and where an instrument is employed to elongate any part,—but if he shall be proved for a month continuously to have exercised the art of a physician, and that made out upon indisputable evidence, however you may conceive the College may have violated their statutes, what improprieties they may have been guilty of, and however ill they may have behaved to licentiates, and whatever disputes there may have been in that learned body, I admit that there must be a verdict against my client. But this, gentlemen, is a penal action; this is what is called a *qui tam* action. These learned physicians bring an action as well for their lord the king as for themselves, and they seek to divide the penalties with the king which they recover from Dr. Harrison; but in order to enable them to maintain the *qui tam* action, they must make out to your satisfaction that Dr. Harrison has practised for a month as a physician.

Now, gentlemen, I will tell you at once what is the dispute between Dr. Harrison and the College of Physicians: it is not whether he may not practise as a physician, but whether he may not exercise his skill in the treatment of spinal complaints. Dr. Harrison, my friend Sir James Scarlett acknowledges,—and Sir Henry Halford would have been very much ashamed if any contrary conduct had been pursued;—Dr. Harrison is a man of liberal education, of profound learning, and great practice in the particular department to which I refer. Would he be afraid to meet Sir Henry Halford? Sir Henry Halford is a most learned physician; but would Dr. Harrison be afraid to undergo an examination before Sir Henry Halford? Sir Henry Halford is not a greater man than Dr. Munro, the professor of anatomy in Edinburgh,—than Dr. Cullen, than Dr. Black, than Dr. Gregory,—than the illustrious medical men who distinguished the University of Edinburgh when Dr. Harrison studied there; and, if he has submitted to an examination under such men, he would not shrink from the examination even of Sir Henry Halford, and all the learning that may now adorn the College of Physicians in London. But, gentlemen, he says they have no right to interfere with his practice, because he has not practised as a physician: he is devoted to one particular disease that afflicts a particular part of the human body, and that there is nothing in the act of Parliament, there is nothing in the charter, which requires that for that purpose he should become a licentiate of the College of Physicians.

Gentlemen, Dr. Harrison practised many years in the county of Lincoln; he then came to London; he has devoted himself to the treatment of that particular complaint. He is, and probably many of you, gentlemen, men of general education and of general reading, may be aware that he is the author of a book entitled "Pathological and Surgical Observations upon Spinal Diseases, illustrated with cases and engravings;" that that is a book of very considerable celebrity, and of very great merit; and that he has devoted his life to the investigation of the evils that arise from a distorted spine. Gentlemen, I say he came to London on purpose that he might devote himself to that particular department of the surgical profession. Does not this entirely correspond with the evidence? I will examine the evidence by and by; but first let me remind you of the well-known distinction in the art which has long subsisted, which was known at the time of Henry VIII., between medicine and surgery. Medicine, gentlemen, is one department; surgery is another. Now, that which is a surgical case is not a physical or a medical case; and what are surgical cases? Why, gentlemen, *ex vi termini*, they are cases in which the hand is employed, in which something is to be done to a particular part of the human frame by means of the hand, or by means of mechanical expedients; and therefore, whenever you see that the case is a surgical case, then that is demonstration that that is not a case which is within the province of a physician.

Now let us see what evidence has been given before you today to show that Dr. Harrison has practised as a physician. He is proved to have been for years practising; this learned body have set all their informers to work; they have industriously employed themselves to find out he has been practising as a physician. They say they have got evidence. Sir James Scarlett referred himself to some other cases, which they have not proved; and the single case which they have brought forward is the case of an unfortunate young lady, of the name of Orton. Now, I ask you,—and I ask you boldly and confidently,—is Dr. Harrison proved to have attended Miss Orton as a physician? I say, boldly and confidently, he did not attend her as a physician. What was her complaint? Her complaint was a disordered spine. Who would you call in for that? Would you call in Sir Henry Halford? No, you would call in Sir Astley Cooper, who is acquainted with anatomy; and if you can find any person who is perfectly conversant with that disorder, you would call in that person: and this young lady had heard of this gentleman, and she calls him in, and he devotes himself to her case, and he employs about half an hour in each day in rubbing her with his hand upon a board, to elongate the spine, and employs an instrument. Did you ever hear of a physician employing a surgical instrument? Who employs surgical instruments? Why, a surgeon. Would Sir Henry Halford, if he were called in,—would he, with his own hand, have operated upon the spine of this unfortunate young woman? would he have employed an instrument for that purpose? Certainly not: he would have said, send for Dr. Harrison,—send for Sir Astley Cooper,—send for a person whose hand is accustomed to this particular employment, and who is in the habit of using the mechanical means which are used for the purpose of curing distortion! Then did Dr. Harrison attend her as a physician or a surgeon? I say he attended her as a surgeon: he performed the functions of a surgeon, and that shews in what capacity he attended. Then there is no other instance given, but this Miss Orton and poor Emma Edwards, the maid-servant. Now, poor Emma Edwards, the maid. What was the matter with her, I did not ask. It might have been a distortion of the spine likewise; although she appears a very good-looking young woman, and I believe it was nothing of that sort; and if it was, she has been cured. My friend said it was something to be taken internally. Now, gentlemen, unless I am very much mistaken, frequently in cases which are purely surgical they prescribe medicine. What case is there in which medicine is not prescribed? I have read surgical books myself, as you have. I have read of the treatment of various disorders; I have read of the treatment of the disease of lues venerea, —there is no impropriety in referring to what I have read upon the subject, —that for a surgeon or a physician the treatment of that disease. I have always understood that was a surgical case, and that the person employed a surgeon who had the misfortune to labour under such a malady. But are there no medicines taken for that? I think you have hydrargyri *quantum sufficit* in the books I have read, and that is signed with the initials of the surgeon; and, gentlemen, the person who receives that prescription from Mr. Cline or Sir Astley Cooper, or any of those who have their levee in the morning, takes it to a neighbouring druggist, and it is made up, and the medicine is taken, and the cure is effected; but, according to this, Mr. Cline might have been summoned before these physicians, and they might have said, "You, Mr. Cline, have practised as a physician, for you not only gave external applications for the disease about which you were consulted, but you prescribed remedies; you said there were some pills to be taken, and therefore that is an internal medicine; and, being an internal medicine, you have prescribed as a physician, and we will levy five pounds a month for every month you have been practising as a physician. Gentlemen, the argument would be just as strong if the case were Mr. Cline's or Sir Astley Cooper's, as if the case were Dr. Harrison's.

Now, in addition to the evidence of Emma Edwards, they have only put in the prescriptions. Why, some internal medicines must have been taken. During the time that the external applications were going on, it was necessary that some internal medicines should be taken. Whatever the object may be, there are medicines that are given internally. One of the most celebrated surgeons of the present day, Mr. Abernethy, says he gives nothing but medicines to be taken internally; and I rather believe, if you went to him for a broken leg, he would say it proceeded from the stomach, and he would write a prescription to be taken; because that which was done formerly, by many medical men, by external applications, is now done by medicines taken into the stomach; and you will say, because a man goes with a broken leg, if he should have some internal remedies given to him, that it is not a surgical case, it is a medical case, because there is physic to be taken: he is practising physic, because there is physic taken; there is physic prescribed, and therefore that is a case of practising physic. Gentlemen, that is the way they reason; but I trust that is not the way you will reason. They can make nothing of Miss Orton; and therefore this College of Physicians, this grave and learned assembly, are to rest their case upon a dose of salts prescribed to Emma Edwards by Dr. Harrison. Gentlemen, really entertaining a respect for the individuals who compose this learned body, I feel compassion for them in their present melancholy situation.

Now, my learned friend Sir James Scarlett, finding he could make nothing at all of his parole evidence, tried to bolster up his case with certain letters that were written by Dr. Harrison. Now, gentlemen, I defy my learned friend to point out one single line in those letters to make out his case; for Dr. Harrison does not at all, in those letters, do more than vindicate what he was doing. He says, " I have a right to do what I am doing, and I will try that question whenever you please. I will admit that practice." But what was he doing? He was attending to spinal complaints; he was devoting himself to that department of the healing art; and when he says he will try it, he is now trying it, and you will observe that the question is, whether attending a spinal case, and labouring with the hand, and employing mechanical means to elongate the spine, is practising as a physician? He was always anxious to try that. If Mr. Roberts had gone to Dr. Harrison, and said that was the admission they wanted, that admission would have been given; but they required an admission contrary to the fact, and therefore that admission was denied: and you will observe what Dr. Harrison says. He says, " I have only to add, in concluding my correspondence, that Messrs. Tennant, Harrison, and Tennant, of Gray's Inn, are my solicitors. To them I refer you in case of your choosing to institute proceedings against me. They are furnished with instructions to give every facility to a legal investigation of your assumed privileges; but they are directed"—(now mind what he says upon this very letter,)—" neither to compromise my rights, nor those of my professional brethren." They would have greatly compromised the rights of Dr. Harrison, and his professional brethren, if they had made the admission that was required. They were ready to make the admission which I now make, and which Dr. Harrison, and those by whom he was advised, were very ready to make, that he has devoted himself to the practice of spinal complaints, and no other.

Now, let us look at the other letter, which I observed Sir James Scarlett was ashamed of, and brought forward with great reluctance. He said he did not intend to read it, and I know he did not, because, from the good feeling which actuates his bosom, he must be sorry that any such correspondence should have passed, or that any such bye-law should have been made by this learned body. Now, what is that bye-law? Gentlemen, it is that, in any case, however urgent and pressing,—in a case of life and death,—no Fellow or Licentiate of the College of Physicians shall have any intercourse with any one who is not a member of the College of Physicians.

*Lord Tenterden.*—There is no such bye-law in evidence.

*Mr. Campbell.*—If my friend will read a letter, he must read the whole of it.

*Lord Tenterden.*—He must read all that it contains.

*Mr. Campbell.*—Then, my lord, it shows there is such a bye-law.

*Lord Tenterden.*—Then you must prove the bye-law.

*Mr. Campbell.*—My lord, I will read the letter.

*Lord Tenterden.*—You must prove the bye-law, or only use it as a matter of argument.

*Mr. Campbell.*—My lord, what Dr. Harrison states in his favor must be taken as well as what he states against himself. " I was not a little surprised, on my return to Quebeck-street last Sunday evening, to learn that you had formally refused to meet me in consultation, because I had not received a licence to practise medicine from the London College of Physicians. As the delicate sufferer was at the time in the greatest possible danger, I leave you to form your own conclusions upon the humanity and propriety of declining to give assistance to an afflicted fellow-creature, in compliance with a capricious and untenable bye-law." What does that refer to? Why it refers to the case of an afflicted fellow-creature, who was in the greatest possible danger, and Dr. Chambers being called upon to consider the case of that fellow-creature;—what the nature of the malady was is not disclosed,—but Dr. Chambers being called upon to meet Dr. Harrison in that case, when a fellow-creature was in the greatest possible danger, refused it, and urged a bye-law of the College of Physicians, that no Fellow or Licentiate could meet, in any case, however pressing, a person who was not either a Fellow or Licentiate. Gentlemen, that accounts for the reluctance of my friend Sir

James Scarlett to bring forward that letter, which I think reflects no credit upon the College of Physicians, and which shews the foundation for the observation which Lord Mansfield made, fifty years ago, upon their bye-laws—that they required a serious revision. Then what does that letter prove? Does it prove any thing? That letter proves nothing except that odious and detestable bye-law of the College of Physicians. It proves nothing for the case of my learned friend Sir James Scarlett; it does not prove that the case there referred to is a case different from those upon which Dr. Harrison had always employed himself; nor does it prove that he had, in a solitary instance, in a case of urgent necessity, when the life of a fellow-creature was at stake,—that he had, even when he was willing to give all the relief he could for the sake of the sufferer, and the family of the sufferer, practised as a physician, or continued to practise as a physician for a month continuously, —that is, the whole time. Therefore, gentlemen, that letter does not assist the case of my friend; it does not prove the allegation which my friend is bound to establish; and therefore that letter, as I shall contend, as well as the parole evidence which has been given, concur in this proposition, that the College of Physicians have wholly failed in making out what they have endeavoured to prove.

Gentlemen, that being the case, I think you cannot hesitate a moment about the verdict which it will be your duty to pronounce. You will go upon the evidence, I allow, without considering the policy or impolicy of the law; but at the same time, as men, you will feel some satisfaction in finding that the College of Physicians are wholly unable to substantiate their odious pretensions, and will dismiss them from this Court with a useful lesson.

Lord Tenterden.—Gentlemen of the Jury; The single question for your consideration is this fact alone: Has it been made out that Dr. Harrison did exercise the faculty of physic during the period mentioned in the declaration? With the expediency of the charter we have nothing to do. We must abide by the law as it exists. The bye-law is of such a nature as to require confirmation by an act of Parliament, which it is alleged to have received. It was properly objected by the counsel, that that which was given to me *as* an act of Parliament, is in fact not one. This point I have reserved for the decision of the whole Court." His lordship then minutely recapitulated the evidence, the substance of which we have given, and afterwards proceeded to comment upon the defence. "It had been said on behalf of the defendant, that the proofs only went to his practising as a surgeon, and that he had a right to do so without incurring the penalty of the bye-law. This was true. But if a man exercises both physic and surgery, then he is within the bye-law. It was very extraordinary that the defendant should contend that he had not practised as a physician at all; that he had merely practised as a surgeon. He had been challenged upon the subject, and was called upon to take up his licence, or to discontinue his practice. What did he say? Not that he did not practise as a physician; but he repeatedly asserted that he had a right to do so, and that the bye-laws could not be maintained. That was the substance of his letters. Dr. Chambers had refused to meet Dr. Harrison, but no physician ever refused to give his advice in company with a *surgeon*. Every expression in the letter from Dr. Harrison to Dr. Chambers asserted the right the defendant had to practise as a physician, without the licence of the College. Taking his letters in connexion with the parole evidence, which went to prove, not that the defendant attended the patient for a complaint which was considered as surgical, but that he also wrote prescriptions, which had been produced, and which were signed by him as a physician would sign them, it was for the jury to say whether they were satisfied he had practised as a physician. If they were of that opinion, their verdict would be for the plaintiffs. But if they were of opinion that he had not acted as a physician, but as a surgeon only, that was not against the law, and their decision would be in favor of the defendant."

The jury, after being absent nearly an hour, found a verdict for the defendant.

The power of the College of Physicians has been several times disputed, by different persons; but we believe that the principal doubts as to the validity of the charter, which were urged by Mr. Campbell, have never before been started. We are aware that the plea founded on the supposition that the act of Parliament never received the royal confirmation, was overruled by Chief Justice Pemberton. (2 *Show.* 166. See also College of Physicians *v.* Huybert, Goodall's Collect. 267.*) But, if the legality of the charter were indisputable, Lord Tenterden would surely have at once determined the question: he would not have thought it necessary to take the opinion of the judges. Neither would Sir James Scarlett have omitted so ready an answer to Mr. Campbell, as that the objections he took had been already set aside, if they ever had been. The case of Bonham, referred to by Sir James Scarlett, and that of Dr. Stanger,† which we have frequently heard adduced as evidence of the power of the College, are not so decidedly to the purpose as they are reported to be. In these cases, the doubts raised by Mr. Campbell were not urged; the legality of the charter was not, we believe, scrutinised. It did not occur to the counsel to dispute it. But it does not follow that, because the charter has been recognised, *sub silentio*, in previous cases, that it is a good and perfect charter. The repeated recognition of an imperfect act cannot make it perfect. In our opinion, therefore, the power of the College of Physicians still remains a moot point.

The motives which influence the decision of a jury, can be known only to themselves; but when we reflect upon the pointed termination of Lord Tenterden's summing-up, we cannot doubt that the verdict went for the defendant because the jury believed he had been proved to act as a surgeon, and not as a physician. We, therefore, differ altogether from the opinion expressed upon this subject by one of our contemporaries.

With respect to the conduct of Dr. Harrison, there is but one opinion. He has gained a verdict, but not upon his original cause. He asserted that he had a right to practise as a physician, and he terminates by showing that he is a SURGEON! Can he reflect upon his very equivocal success with any gratification? or has he a right to complain if we accuse him of the most direct tergiversation? To our feelings, defeat would have been much more palatable than such a triumph, sweetened even as it was by the puffs so adroitly introduced by the well-instructed counsel. In a word, Dr. Harrison entered the cause as a volunteer: he abandoned it as a deserter. Well may we ask—

Quid dignum tanto feret, hic promissor hiatu?

* Medical Jurisprudence, by Paris and Fonblanque, vol. i. *note* p. 17.
† 7 Term Rep. 282.

### METEOROLOGICAL JOURNAL,

*From May 20th, to June 20th, 1828.*

By Messrs. HARRIS and Co. Mathematical Instrument Makers, 50, High Holborn.

| June | Rain gauge. | Moon. | Thermom. | | | Barometer. | | De Luc's Hygrom. | | Winds. | | Atmospheric Variations. | | |
|---|---|---|---|---|---|---|---|---|---|---|---|---|---|---|
| | | | 9 A.M. | MAX. | MIN. | 9 A.M. | 10P.M. | 9 A.M. | 10P.M. | 9 A.M. | 10P.M. | 9 *a.m.* | 2 *p.m.* | 10 *p.m.* |
| 20 | | | 62 | 73 | 57 | 29.85 | 29.84 | 80 | 83 | SW | W | Cloudy | Cloudy | Cloudy |
| 21 | .33 | ☽ | 60 | 68 | 56 | .73 | .75 | 96 | 82 | WNW | W | Rain | Fine | Fine |
| 22 | .47 | | 57 | 75 | 55 | .70 | .65 | 87 | 82 | SW | WSW | —— | Rain | —— |
| 23 | | | 57 | 75 | 55 | .90 | 30.13 | 80 | 82 | NW | NNW | Fine | Fine | —— |
| 24 | | | 67 | 72 | 58 | 30.13 | .13 | 83 | 76 | N | N | —— | —— | —— |
| 25 | | | 71 | 75 | 61 | .15 | .18 | 81 | 77 | WNW | NW | —— | —— | —— |
| 26 | | | 72 | 74 | 64 | .18 | .14 | 79 | 74 | N | SW | —— | —— | —— |
| 27 | | | 74 | 80 | 60 | .10 | .18 | 87 | 78 | SW | SE | —— | —— | —— |
| 28 | | | 74 | 79 | 60 | 29.94 | 29.90 | 90 | 75 | E | E | Cloudy | —— | —— |
| 29 | | ○ | 72 | 77 | 61 | .83 | .81 | 100 | 76 | ESE | SE | —— | —— | —— |
| 30 | | | 70 | 76 | 65 | .85 | .81 | 79 | 78 | ESE | SW | Fine | —— | —— |
| July 1 | | | 72 | 75 | 56 | .80 | .77 | 83 | 80 | W | WSW | —— | —— | —— |
| 2 | | | 71 | 76 | 74 | .75 | .73 | 96 | 47 | W | W | Cloudy | —— | Cloudy |
| 3 | | | 76 | 79 | 72 | .76 | .77 | 51 | 45 | W | W | —— | —— | Fine |
| 4 | .18 | ☾ | 75 | 76 | 66 | .70 | .75 | 74 | 43 | SSW | W | —— | Rain | Cloudy |
| 5 | | | 72 | 74 | 77 | .72 | .71 | 46 | 45 | W | W | Fine | Fine | Fine |
| 6 | | | 70 | 70 | 60 | .73 | .72 | 43 | 46 | W | NW | Cloudy | Cloudy | Fine |
| 7 | | | 73 | 76 | 61 | .74 | .70 | 55 | 45 | W | SE | —— | Fine | —— |
| 8 | | | 76 | 78 | 62 | .60 | .78 | 66 | 48 | ESE | SE | —— | Show'ry | Show'ry |
| 9 | .47 | | 77 | 68 | 63 | .41 | .44 | 65 | 65 | NW | N | Rain | Fine | Fine |
| 10 | .70 | | 72 | 70 | 61 | .50 | .50 | 58 | 50 | N | W | —— | Cloudy | Cloudy |
| 11 | | | 68 | 73 | 68 | .83 | .72 | 60 | 55 | WNW | SE | —— | Rain | Rain |
| 12 | .72 | ● | 73 | 65 | 55 | .44 | .40 | 66 | 51 | NW | NW | —— | Rain | Fine |
| 13 | | | 61 | 64 | 55 | .23 | .40 | 50 | 49 | WNW | NW | —— | Cloudy | Fine |
| 14 | .20 | | 64 | 67 | 56 | .42 | .40 | 49 | 47 | W | WNW | Fine | Rain | Rain |
| 15 | | | 61 | 66 | 55 | .35 | .45 | 47 | 48 | W | W | Cloudy | Cloudy | Fine |
| 16 | | | 64 | 72 | 59 | .61 | .64 | 48 | 49 | NNW | W | Cloudy | Fine | —— |
| 17 | .42 | | 73 | 72 | 63 | .58 | .60 | 49 | 50 | WSW | W | Rain | —— | —— |
| 18 | | | 69 | 73 | 61 | .50 | .42 | 50 | 50 | SW | WSW | Cloudy | —— | —— |
| 19 | | | 68 | 70 | 58 | .50 | .47 | 50 | 50 | WNW | W | —— | —— | Fair |

The quantity of Rain fallen in the month of June, was 3 inches and 49.100ths.

